# Inequalities in Poverty and Income between Single Mothers and Fathers

**DOI:** 10.3390/ijerph17010135

**Published:** 2019-12-24

**Authors:** Yuan-Chiao Lu, Regine Walker, Patrick Richard, Mustafa Younis

**Affiliations:** 1 Division of Diagnostic Imaging and Radiology Children’s National Hospital, Washington, DC 20010, USA; yclu@vt.edu; 2 Preventive Medicine and Biostatistics Uniformed Services University of the Health Sciences, Bethesda, MD 20814, USA; regine.walker.ctr@usuhs.edu (R.W.); patrick.richard@usuhs.edu (P.R.); 3 Department of Health Policy and Management, School of Public Health, Jackson State University, Jackson, MS 39217, USA

**Keywords:** single parent, income, poverty, Panel Study of Income Dynamics

## Abstract

Background: The American family structure has changed in the past few decades due to a rise in the divorce rate and unmarried women with children. Research suggests a salary disparity between men and women, especially for those women after pregnancy. However, these studies were confined to individuals within traditional families, and there is a lack of information of income disparity and poverty status between single mothers and fathers. The current study explored the disparities in single-parent families based on the household income and the poverty status using a set of nationwide censor data. Methods: The current study used data from the 2011 and 2013 Panel Study of Income Dynamics (*N* = 1135). Multivariate regression models were used in the analysis. Results: The demographic characteristics of the weighted population showed that taxable income, total income, and poverty status were higher for single fathers than mothers, while non-work income was higher for single mothers than fathers. Single mothers were much more likely to be at the crisis category than single fathers. Multivariate analyses showed that gender, age, marital status, years of experience, and geographic region had effects on taxable income, and only gender, marital status, and region had effects on poverty status. Conclusions: The results suggest that vulnerable group of single mothers was acknowledged according to income and poverty status. Age, marital status, years of experience, and region would be the critical factors for predicting the income and poverty status for single parenthood.

## 1. Introduction

Single parenthood has been a major public policy issue in the United States since the 1960s [1]. In 2012, approximately 21 million children or 28% of all children in the US, lived with one parent [2]. Among the 11.6 million single parents living with their children in 2009, 9.9 million were single mothers and 1.7 million were single fathers [3]. Furthermore, single-parent families in particular have experienced a deterioration in their economic well-being following the recession of 2007–2009, and have remained in a lower state of well-being post-recession compared to pre-recession [2]. Single-mother families and single-father families also had median incomes that were less than they had been in 2000 (in 2008 constant US dollars) [4]. This could be crucial given that several studies have found that children from low-income families perform worse on a set of academic achievement indicators and have more behavior problems than children from higher income families [5,6,7]. Although the prevalence of poverty and financial disparity between single mothers and single fathers has been discussed in the literature, a thorough understanding of the factors that may impact this is still needed. 

Several factors may have an impact on the socio-economic status of single-parent families. Racial/ethnic differences have been raised as a potential determinant of the low socio-economic status of single-parent families [1,8,9]. Furthermore, other studies have focused on the effect of a mother’s disability status or the child’s health, such as disability or emotional and behavioral health problems, on the mothers’ participation in the work force [10,11,12,13,14]. Additionally, a study by Ziol-Guest [15] found that single fathers made different purchasing decisions compared to single mothers and married parents. For example, single fathers spent more on food consumed outside of the home, alcohol, and tobacco products and less on children’s education than single mothers and married parents [15].

The disparity of income and poverty status between single mothers and single fathers was investigated and discussed by Kramer et al. [1]. They explored the disparity in taxable income and poverty status between employed single mothers and single fathers using the US Census of Population and Housing, Public Use Microdata Sample from 1990, 2000, and 2010. They found that single mothers were more likely to be in poverty than single fathers. However, while the study was very informative, all of the explanatory variables (gender, race, number of children, age, marital status, work hours per week, weeks worked in the previous year, occupation score, and education) used in the analyses were statistically significant, leaving policy-irrelevant trivial associations. This may be due to a large sample size (*N* = 219,743 for 1990, *N* = 273,463 for 2000, and *N* = 59,099 for 2010, total *N* = 554,633). It has been suggested that, in very large samples, *p*-values can be close to zero, and thus solely relying on *p*-values can lead to claims of support for results of no practical significance [16]. In addition, in large sample sizes, small effects can be indicated as significant and confidence intervals can be very narrow [17,18]. Thus, the null hypothesis can often be rejected in cases with very large sample sizes [18].

In the current study, two objectives were tested using a more appropriate sample size to identify the factors for differentiating the socio-economic status of single-parent families. The first objective was to identify the significance of the predictors used by Kramer et al. [1] by using a proper sample size with a similar model specification. The sample size calculation is provided in Section 2.1. The second objective was to improve the model specification to better understand the determinants of poverty status and income of single mothers and single fathers. The improved model specification involves changing the form of the predictors (e.g., adding the squared of age to the regression models) to better capture the nonlinear relationship between some of the independent variables and the dependent variables. It is hypothesized that a dataset with a smaller sample size and the modified regression model specification may better indicate the significance and practicability of the predictors. 

## 2. Methods

### 2.1. Data Source and Participants

Data from the Panel Study of Income Dynamics (PSID) collected in 2011 and 2013 were pooled and utilized in this study [19,20]. The PSID is a nationally representative sample of individuals living in families in the US [21,22,23,24]. This longitudinal survey collects socioeconomic and demographic data (e.g., employment and marital status). 

The selection of subjects was based on the study by Kramer et al. [1]. The sample consisted of working, single-parent head of households 18–64 years old with positive taxable income. There was only one parent in the household and the parent was the only adult (18 years old or older) in the household. Multigenerational and cohabiting households were removed. Subjects held employment and received a salary in the prior year. Subjects were considered employed if they reported employment during 1–52 weeks last year. The number of observations in the combined 2011 and 2013 PSID data in the analytic sample consisted of 1135 single-parent headed households in which 86.5% (*N_M_* = 981) were single-mother households and 13.5% (*N_F_* = 154) were single-father households. The sampling weights for the number of subjects that each observation represents in the population were applied, resulting in the weighted sample size of 13,808 households (female: 11,288; male: 2520). The weighted sample size was used for subsequent demographics calculation and multivariate regression analyses. Although the current study did not use the same years as Kramer et al. [1], time fixed effects addressed any changes over time that may be associated with the outcomes.

The sample size was verified using the following formula suggested by Eng [25]:

(1) $$N=\frac{4\sigma^{2}{\left(Z_{crit}+Z_{pwr}\right)}^{2}}{D^{2}}$$

where N is the total sample size, σ is the assumed standard deviation of each group (assuming equal for both groups), $Z_{crit}$ is the desired significance criterion, $Z_{pwr}$ is the desired statistical power, and D is the minimum expected difference between the two means. Poverty status was used as the index to calculate the sample size. σ was set as 15, which was the standard error (S.E.) of the full sample. A significance criterion of 0.05 ($Z_{crit}$ = 1.96) and power of 0.95 ($Z_{pwr}$ = 1.645) were chosen. To discriminate a 1-unit difference (D = 1) of poverty status between single mothers and single fathers, a sample size of 11,696 was sufficient. This analytic sample size, 11,696, was close to the weighted sample size of 13,808 households used in the current study.

## 3. Measures

### 3.1. Objective 1 

The first objective was to identify the significance of the predictors by implementing multivariate regression analyses with a proper sample size using a similar model specification as that by Kramer et al. [1]. Two dependent variables were calculated: (1) taxable income reported from the corresponding preceding years, i.e., 2010 and 2012; and (2) poverty status. Taxable income included the single parent’s income from assets, earnings, and net profit from a farm or business. A logarithmic transformation was used to account for skewness in the distribution of income [26,27,28]. It is important to note that only positive values of non-work income were logarithmic transformed and analyzed in the multivariate regression models for the full sample for the weighted population. 

Poverty status was calculated as taxable income divided by poverty threshold, and it was treated as a continuous variable in the multivariate regression analyses. The poverty threshold criterion was from the US Census Bureau definition based on family size and the number of individuals in the family under the age of 18 [29]. Furthermore, similar to Kramer et al. [1], the four poverty levels proposed by Bauer et al. [30] were used to further portray poverty status. Families at or below 100% poverty status are in crisis, greater than 100% and below/equal to 150% poverty status are at risk, greater than 150% and below/equal to 200% poverty status are safe, and greater than 200% poverty status are thriving. To account for currency inflation, the income from 2010 was adjusted to the amount in 2012 using the Consumer Price Index [31]. The cumulative rate of inflation from 2010 to 2012 was 5.3%. 

In the first objective, similar model specifications as Kramer et al. [1] were used. The key independent variable was the gender of the single parent. Single-parent families were coded as 1 if the head of household was a single mother or 0 if the head of household was a single father. Control variables included the head of household’s race, age, marital status, number of hours that the head of household worked, number of weeks that the head of household worked, non-work income, occupational income score, and education. Race/ethnicity was categorized into White, Black, and Other race and was treated as a categorical variable (reference: White). The Other race category included Hispanic, Asian, American-Indian or Alaska Native, and Native Hawaiian or Pacific Islander. Age was treated as a continuous variable ranging from 18 to 64 years old. Marital status was categorically coded by Divorced, Widowed, Separated, and Never married (reference: Divorced). The number of hours that the head of household worked on average during the week in the corresponding previous year was included as a continuous variable. The number of weeks that the head of household worked in the corresponding previous year was classified into six groups: 1–13 weeks, 14–26 weeks, 27–39 weeks, 40–47 weeks, 48–49 weeks and 50–52 weeks. Non-work income, the sum of social security income, welfare income, supplemental security income, interest income, dividend income, and retirement income, was a predictor in Objective 1. In our study, we collected all accessible income sources rather than the taxable income in PSID data for the non-work income, and these non-work income sources were close to the list of non-work income data used by Kramer et al. For the occupational income score, data from the PSID was used, but similar to Kramer at al. [1], the Integrated Public Use Microdata Series was used to generate the scores (3 (low occupational score) to 80 (high occupational score) based on income). For education, a high school degree or less was coded as 1 and higher than high school was coded as 0. Household variables included geographic region and the number of children. Geographic region was categorically coded as Northeast, North central, South and West (reference: Northeast). The number of children (under 18 years old) in the household was included in the analyses as a continuous variable. Based on Kramer et al. [1], the number of children and non-work income were not included in the model in cases where the dependent variable was poverty status because poverty status is calculated based on the income and the number of dependents that single parents have. The interaction effects between gender and number of children, gender and work hours per week, gender and occupational income score, and gender and education were also included in the analyses. Dummy variables indicating the years of 2011 and 2013 were also included in the regression models to capture any time trends in the dependent variables.

### 3.2. Objective 2

The second objective was to improve the model specification to better examine the determinants of poverty status and income of working single mothers and single fathers. In addition to the two dependent variables used in Objective 1, two additional dependent variables were considered for Objective 2: non-work income and total income. While non-work income was treated as a predictor in Objective 1, it was considered as a dependent variable in Objective 2. This is similar to Zhan and Pandey’s study [32] that showed non-work income is not a predictive variable for taxable income; therefore, taxable income and non-work income were treated as dependent variables. For Objective 2, non-work income additionally included transfer income. Transfer income was the income from the government in the form of benefits or subsidies, excluding the social security income. Total income was the sum of taxable income and non-work income. For calculating poverty status for Objective 2, instead of using taxable income as described in Objective 1, poverty status was calculated as *total* income divided by poverty threshold, based on the definition of total income used to compute poverty status by the US Census Bureau [33]. 

The key independent variable was the gender of the single parent. The category Hispanic was included in the race variable. Race/ethnicity was categorized into White non-Hispanic, Black non-Hispanic, Hispanic, and Other race (Asian, American-Indian or Alaska Native, and Native Hawaiian or Pacific Islander). The number of children was log transformed because the data were positively skewed [14,34]. Age squared was added to the regression models for age in order to capture the nonlinear relationship between age and income/poverty status [13,14]. The number of work hours per week and number of weeks worked per year were excluded in the regression models in Objective 2 since they are collinear with income/poverty status [12]. The subject’s years of education was treated as a continuous variable, ranging from 1 to 17 years where the range of 1–6 represents the actual grade of school completed and 17 represents receiving education beyond college [12]. Years of education squared was added to the regression models to capture the nonlinear relationship between years of education and income/poverty status. Years of experience and its squared term were added to the models according to the Mincer equation [35]. The occupational income score was not considered in Objective 2 because it is collinear with income/poverty status. The specifications of marital status, geographic region, and dummy variables for year were kept the same as for Objective 1. The interaction effects between gender and other independent variables were excluded in the regression models [9,32].

## 4. Statistical Analysis

### 4.1. Summary Statistics 

Weighted means, proportions, and linearized S.E.s of all dependent and independent variables included in the analysis were calculated for the full sample. Adjusted Wald tests were used to test the mean differences for the continuous variables and chi square tests were used to test the statistical significance of proportions for the categorical variables between single mothers and single fathers at the 0.05 significance level. 

### 4.2. Multivariate Regression Analysis 

Two sets of multivariate regression models were used in the analysis. For comparison, the first set of model specifications to predict taxable income and poverty status for Objective 1 were very similar to those used by Kramer et al. [1]. For Objective 2, which aimed to predict taxable income, non-work income, total income, and poverty status, we improved on the model specifications by taking into account the nonlinear relationship between some variables such as age, years of education, and years of experience, as has been found in the literature [35]. We also excluded work hours from the models as this variable has been found to be collinear with income [35]. The following equation was used to estimate the income variables:

(2) $$\log M_{i}=\beta_{0}+\beta_{1}\cdot G+\beta \cdot X_{i}+\varepsilon_{i}$$

where $M_{i}$ represents taxable income for Objective 1 and taxable income, non-work income, or total income for Objective 2. G represents the gender of the single parent. $X_{i}$ is a vector of the independent variables. The parameters $\beta_{0}$, $\beta_{1}$, and β are to be estimated and $\varepsilon_{i}$ is the random error term.

A similar approach was used to estimate poverty status:

(3) $$S_{i}=\delta_{0}+\delta_{1}\cdot G+\delta \cdot X_{i}+\upsilon_{i}$$

where $S_{i}$ represents poverty status. The parameters $\delta_{0}$, $\delta_{1}$, and δ are to be estimated and $\upsilon_{i}$ is the random error term.

The results of the multivariate regression analysis are presented for the full sample for Objective 1 and for the full sample for Objective 2. Oversampling of immigrant and minority households and attrition in the PSID were accounted for by using family weights [19,20]. In this case, a robust variance estimation technique was used to adjust for design characteristics so that variances, standard errors and confidence intervals are correct [19,20]. Statistical analyses were conducted using Stata 14 [36].

## 5. Results

### 5.1. Summary Statistics

The demographic characteristics of the weighted population were calculated for the continuous and categorical variables (Table 1). Single fathers’ average taxable income was higher than that of single mothers’ ($56,458 vs. $35,287, *p* < 0.05). On the other hand, the average non-work income for single mothers was higher than that of single fathers ($4879 vs. $1320, *p* < 0.01). Total income was higher for single fathers than single mothers ($57,778 vs. $40,165, *p <* 0.05). Poverty status (taxable income divided by poverty threshold) was higher for single fathers than single mothers even after using a better model specification for Objective 2 (total income divided by poverty threshold) (*p* < 0.05). 

**Figure 1:**
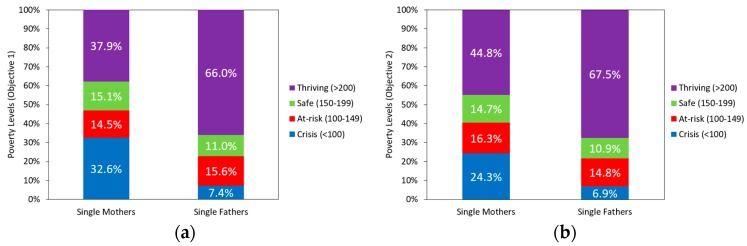
Poverty levels of single mothers and single fathers: (**a**) poverty status is taxable income divided by poverty threshold for Objective 1; and (**b**) poverty status is total income divided by poverty threshold for Objective 2.

Furthermore, the proportions of the weighted population for poverty levels by gender are shown in Figure 1. For Objective 1, the results for poverty status showed that 32.6% of single mothers were at the crisis category, while 7.4% of single fathers were at the crisis category. When total income is taken into account for Objective 2, the results for poverty status showed that 24.3% of single mothers were at the crisis category, while 6.9% of single fathers were at the crisis category. Single mothers were much more likely to be at the crisis category than single fathers.

### 5.2. Multivariate Results

#### 5.2.1. Objective 1

The first objective was to compare the multivariate regression results from Kramer et al. [1] by using a similar regression model specification but a proper sample size. It is recalled that each dependent variable (taxable income and poverty status) was regressed on the independent variables for the full sample in Objective 1. The comparisons of the multivariate regression coefficients are shown in Table 2. Unlike Kramer et al. [1], who showed that all of the predictors were significant, only age, work hours per week, weeks worked last year, occupational income score, education, and geographic region (North central) were significant factors when predicting taxable income at the 5% significance level. In addition, only race (Other race), age, marital status (Separated), work hours per week, weeks worked last year, and geographic region (North central) were significantly associated with poverty status at the 5% significance level. Contrary to the findings of Kramer et al. [1], the interaction terms were not significant for either regression model at the 5% significance level. The results show, that with a proper sample size, not all of the variables from Kramer et al. [1] are significant, suggesting that very large sample size may be associated with significance of very small and trivial effects.

#### 5.2.2. Objective 2

The second objective was to improve the model specification used by Kramer et al. [1]. It is recalled that each dependent variable (log-transformed taxable income, non-work income, total income, and poverty status) was regressed on the independent variables for the full sample. 

In the full sample model (Table 3), the dependent variables were regressed on all explanatory variables, including gender. The results showed that single mothers had less taxable income but received higher non-work income than single fathers (*p* < 0.001). Total income was higher for single-father families than for single-mother families (*p* < 0.001). Single mothers had a lower poverty status than single fathers (*p* < 0.01). The number of children was positively associated with non-work income (*p* < 0.05). Age was positively associated with taxable and total income (*p* < 0.01). However, as expected, this relationship was not linear as age squared was negative, meaning that there is a diminishing marginal return in the association between age and taxable and total incomes. For marital status, significant differences were observed when comparing separated to divorced for the taxable income, total income, and poverty status models. Specifically, single parents who were separated had lower taxable income, total income, and poverty status than those who were divorced (*p* < 0.05). Years of experience was a highly significant factor when predicting taxable and total income (*p* < 0.001). Finally, geographic region was a predictive factor for taxable income, total income, and poverty status. Compared to those who resided in the Northeast, single parents who lived in the North central region or the South had a lower taxable income, total income, and poverty status. 

## 6. Discussion

This study conducted a comprehensive analysis to evaluate the association between the gender of the head of household and income and poverty status in single-parent families while controlling for an extensive set of variables based on a modified Mincerian approach [35]. There were two objectives in our study—one was to revisit Kramer’s results and compare them to PSID data, and the other one was to provide a new perspective of the multivariate regression model specifications for analyzing the PSID data. These two objectives were serving different critical arguments for modeling the data. While Objective 1 offers essential advice for processing and interpreting survey data as well as the sample size issues, Objective 2 provides informative results regarding different income types. As expected, significant factors can be identified from a relatively smaller dataset than that used by Kramer et al. [1]. As shown in several previous studies [16,17,18], a large sample size may be associated with significant observations, but with very small effects, limiting the conclusions that can be made. It does not imply that a smaller sample size is better than a larger sample size, but, when having a larger sample size, it is harder to understand the implications of the results because everything is statistically significant. Furthermore, with an improved model specification, it was found that only gender, age, age squared, marital status, years of experience, years of experience squared, and geographic region were associated with taxable income. Only gender, marital status, and geographic region were associated with poverty status. In addition, gender was not significant when using a similar model as Kramer et al. [1], but was for the improved model when predicting taxable income and poverty status. 

The sample size for the current study was verified using a formula suggested by Eng [25] and it was found that a weighted sample size of 11,696 observations was sufficient. This is close to our weighted sample size of 13,808. In addition to addressing the sample size, the regression models in the current study were modified and improved [11,12,32,35]. First, we squared age to capture the nonlinear relationship between age and income/poverty status and included it in the models [11,12]. Second, work hours per week and weeks worked last year were not considered because they are collinear with income/poverty status [13,14]. Indeed, the results from the original model specification (Table 2) show that work hours per week and weeks worked last year were significant when predicting taxable income and poverty status. Third, years of experience and its squared term were added to the improved model based on the Mincer equation [35]. The Mincer equation explains income as a function of education and experience [35]. Fourth, years of education was used to replace the dichotomy of the education variable used by Kramer et al. [1] (i.e., high school degree or less vs. some college education or more) and years of education squared was included to capture the nonlinear relationship between years of education and income/poverty status [12]. Lastly, no interaction terms of the predictors were added to the models because the interactions were not significant. 

The significant independent variables found in this study for predicting the taxable and total income include the age and its square, Separated (vs. Divorced), Years of experience and its square, and North central and South (vs. Northeast) (Table 3). According to the CPS microdata from the University of Minnesota’s Minnesota Population Center, the 50th percentiles of the taxable income were $13,520, $35,490, and $42,696 for age groups 18–24, 30–34, and 50–54, showing the nonlinear, positive correlation between the age and income [37]. Our study also found that the separated single parents have lower taxable and total income when compared to divorced single parent. This could be due to the fact that legal separation allows for the retention of health care and other benefits including certain social security benefits that terminate with a divorce, and spouses may still be responsible for the debt of the other in a legal separation, unlike a divorce where the debts are handled during the dissolution process. The years of experience was typically correlated with age [4] and therefore higher income was expected. The regional differences of taxable income and total income of single-parent families corresponded well to the nationwide data [4] where South and North central have less average salary than Northeast while West has comparable salary level when compared to Northeast. For the non-work income, we found that number of children was the only significant independent variable (Table 3). As expected, this is because the non-work income consisted of mainly social support and welfare income, and therefore higher number of children would lead to higher non-work income.

The findings of the current study comparing single mothers and single fathers matched well with previous studies. The current study showed that the average total income and poverty status of single mothers were lower than the average US household, a finding consistent with previous observations [38]. Livingston et al. found that single fathers are slightly older than single mothers with 18% and 23%, respectively, under the age of 30 [39]. The current study showed that the mean age for single mothers was 36.44 years and for single fathers was 38.56 years, and that 24.6% of single mothers and 13.5% of single fathers were under the age of 30. In addition, Livingston et al. found that single-father householders were more likely to be white than single mother householders (56% and 45%, respectively) and single fathers were less likely to be black (15%) than single mothers (28%) [39]. In our study, these proportions were slightly larger for single fathers and single mothers (68% and 50% for white and 19% and 34% for black, respectively). Furthermore, the mean years of education for the poverty levels (total income divided by poverty threshold) crisis, at-risk, safe, and thriving for single mothers were 12.17, 12.79, 13.75, and 14.48, respectively. This result was consistent with Grall’s findings that the highest poverty rates were found among single mothers who had less than a high school education [40]. With the above evidence, it could be concluded that the data from the PSID might serve as a reliable source in the analysis of single parenthood and social-economic status.

**Figure 2:**
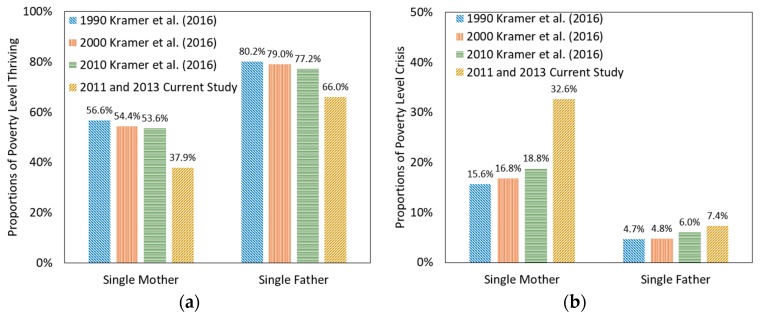
Proportions of single mothers and fathers at (**a**) crisis and (**b**) thriving poverty categories (1990–2013).

The proportions of the weighted population for the four poverty levels (crisis, at-risk, safe, and thriving) followed the trend observed by Kramer et al. [1] (Figure 2). They observed that 15.6–18.8% of single mothers and 4.7–6.0% of single fathers were at the crisis poverty category from 1990 to 2010. In the current study, a larger proportion of single mothers and single fathers were observed at this category (32.6% and 7.4%, correspondingly). Furthermore, Kramer et al. found that 53.6–56.6% of single mothers and 77.2–80.2% of single fathers were at the thriving category from 1990 to 2010. The current study showed that 37.9% of single mothers and 66.0% of single fathers were observed at the thriving category. As commented by Kramer et al., both single mothers and fathers had a decline in their financial status over time, with those in crisis increasing and those thriving decreasing. 

There are a few limitations in this study. First, our selected samples from the PSID included only subjects with positive taxable income to make comparisons with Kramer et al. [1]. A comparison of the financial status of single mothers and single fathers that incorporates unemployment should be further investigated. Second, while the current study conducted an assessment of a wide range of independent variables to compare to the study by Kramer et al. [1], other essential predictors such as the health status of single parents and their net worth/home equity should also be considered in future studies. These predictors should be included in regression models to gain a more comprehensive understanding of the differences between single mothers and single fathers.

## 7. Conclusions

In summation, the current study found significant differences between single mothers and single fathers in terms of taxable income, non-work income, total income, and poverty status. The results suggest that vulnerable group of single mothers was acknowledged according to income and poverty status, and the evaluation of income and poverty for single parenthood could provide reliable evidence to policymakers. Future studies should utilize proper sample sizes and appropriate functional forms in the evaluation of income and poverty status for single parenthood to deduce convincible suggestions for the policymakers.

## Figures and Tables

**Table 1:** Weighted means/proportions (M/P) and standard errors (S.E.) of dependent and independent variables (pooled Panel Study of Income Dynamics (PSID) 2011 and 2013) by single-parent status.

Variables	Full Sample *N* = 1135		Single Mothers *N_M_* = 981		Single Fathers *N_F_* = 154		*p*-Value
	M/P	S.E.	M/P	S.E.	M/P	S.E.	
Taxable income ($)	39,150	2401	35,287	2265	56,458	7214	0.01
Non-work income ($)	4229	552	4879	643	1320	263	0.00
Total income ($)	43,379	2612	40,165	2569	57,778	7233	0.02
Poverty status (%) (Objective 1)	229	14	204	13	343	45	0.00
Poverty status (%) (Objective 2)	253	15	231	15	351	45	0.01
Age (years)	36.83	0.52	36.44	0.57	38.56	0.98	0.05
Work hours per week	39.48	0.60	38.54	0.70	43.70	1.19	0.00
Categorized weeks worked last year	4.93	0.06	4.86	0.07	5.26	0.12	0.01
Occupation score	30.85	0.96	31.38	1.11	28.48	1.72	0.17
Number of children	1.66	0.04	1.72	0.05	1.43	0.07	0.00
Years of education	13.53	0.12	13.54	0.13	13.51	0.25	0.93
Years of experience	5.18	0.31	5.13	0.32	5.43	0.77	0.71
Race							
White	0.54	0.05	0.50	0.06	0.68	0.05	0.01
Black	0.31	0.05	0.34	0.05	0.19	0.05	0.01
Hispanic	0.13	0.02	0.14	0.03	0.08	0.03	0.06
Other	0.02	0.01	0.02	0.01	0.06	0.03	0.17
Marital status							
Divorced	0.43	0.03	0.41	0.04	0.52	0.05	0.10
Widowed	0.02	0.01	0.01	0.01	0.04	0.02	0.32
Separated	0.12	0.01	0.11	0.01	0.17	0.05	0.21
Never married	0.43	0.04	0.47	0.04	0.26	0.05	0.00
Education							
High school degree or less	0.40	0.03	0.40	0.03	0.37	0.06	0.62
Higher than high school	0.60	0.03	0.60	0.03	0.63	0.06	0.62
Region							
Northeast	0.16	0.04	0.15	0.04	0.23	0.05	0.10
North central	0.28	0.03	0.29	0.03	0.22	0.05	0.20
South	0.34	0.03	0.35	0.04	0.31	0.05	0.43
West	0.22	0.05	0.22	0.05	0.24	0.04	0.57
Year							
2011	0.49	0.02	0.50	0.02	0.45	0.04	0.20
2013	0.51	0.02	0.50	0.02	0.55	0.04	0.20

Note: The poverty status for Objective 1 is taxable income divided by poverty threshold, while the poverty status for Objective 2 is total income divided by poverty threshold.

**Table 2:** Comparison of multivariate regression coefficients of independent variables predicting log taxable income and poverty status between Kramer et al. [1] and the current study (full sample).

Multivariate Regression	Log Taxable Income				Poverty Status			
Variables	Kramer et al. (Kramer 2016)			Current Study	Kramer et al. (Kramer 2016)			Current Study
Year	1990	2000	2010	2011–2013	1990	2000	2010	2011–2013
				Coef.(S.E.)				Coef.(S.E.)
(Male)								
Female	−0.55 ***	−0.44 ***	−0.48 ***	−0.11	−63.46 ***	−42.31 ***	−30.67 ***	57.81
				(0.61)				(145.63)
(White)								
Black	0.04 ***	0.01 ***	−0.03 ***	−0.07	−13.01 ***	−14.69 ***	−17.50 ***	−29.24
				(0.09)				(20.16)
Other Race	−0.05 ***	−0.09 ***	−0.08 ***	−0.09	−23.91 ***	−24.45 ***	−18.76 ***	−60.85 *
				(0.10)				(25.47)
Number of children	0.01 ***	0.01 ***	0.01 **	0.04				
				(0.07)				
Age	0.01 ***	0.01 ***	0.01 ***	0.02 ***	5.67 ***	5.69 ***	4.79 ***	6.30 **
				(0.01)				(1.89)
(Divorced)								
Widowed	−0.12 ***	−0.11 ***	−0.08 ***	−0.28	5.82 ***	6.59 ***	6.90 ***	−64.43
				(0.24)				(32.65)
Separated	−0.09 ***	−0.06 ***	−0.10 ***	−0.16	−22.54 ***	−23.07 ***	−29.53 ***	−49.14 *
				(0.09)				(23.56)
Never Married	−0.13 ***	−0.11 ***	−0.11 ***	−0.08	−4.98 ***	−7.00 ***	−15.00 ***	11.26
				(0.12)				(34.44)
Work hours per week	0.01 ***	0.02 ***	0.02 ***	0.04 **	1.54 ***	2.16 ***	2.81 ***	6.98 *
				(0.01)				(3.16)
Categorized weeks worked last year	0.27 ***	0.25 ***	0.29 ***	0.23 ***	22.27 ***	19.89 ***	20.77 ***	12.15 *
				(0.03)				(4.84)
Non-work income	−0.00 ***	−0.00 **	−0.00 ***	0.00				
				(0.00)				
Occupation score	0.02 ***	0.02 ***	0.02 ***	0.01 *	2.94 ***	2.79 ***	3.44 ***	3.92
				(0.00)				(2.15)
(Higher than high school)								
High school degree or less	−0.22 ***	−0.20 ***	−0.26 ***	−0.33 *	−40.07 ***	−35.72 ***	−46.80 ***	−85.86
				(0.12)				(56.83)
Gender × number of children	−0.05 ***	−0.04 ***	−0.03 ***	−0.06				
				(0.07)				
Gender × working hours	0.01 ***	0.01 ***	0.01 ***	0.00	0.56 ***	0.58 ***	0.50 ***	−2.99
				(0.01)				(3.33)
Gender × occupation score	−0.01 ***	−0.01 ***	−0.01 ***	−0.00	−0.11 ***	−0.31 ***	-−0.71 ***	−1.33
				(0.01)				(2.39)
Gender × high school or less	−0.08 ***	−0.10 ***	−0.07 ***	−0.00	−4.60 ***	−11.95 ***	−10.09 ***	20.11
				(0.15)				(58.93)
(Northeast)								
North central	NR	NR	NR	−0.23 **	NR	NR	NR	−57.92 *
				(0.09)				(23.68)
South	NR	NR	NR	−0.12	NR	NR	NR	−38.77
				(0.09)				(24.18)
West	NR	NR	NR	0.04	NR	NR	NR	7.24
				(0.11)				(31.62)
(Year 2011)								
Year 2013				−0.03				−17.39
				(0.05)				(10.50)
Constant	7.23 ***	7.44 ***	7.11 ***	6.83 ***	−163.66 ***	−186.27 ***	−209.17 ***	−293.74
				(0.67)				(155.49)
*N*	219,743	273,463	59,099	1135	219,743	273,463	59,099	1135
Adjusted R^2^	0.499	0.474	0.544	0.563	0.473	0.463	0.475	0.364

* *p* < 0.05; ** *p* < 0.01; *** *p* < 0.001. Note: NR, Not reported. Coef., Coefficient. S.E., Standard error. Numbers in brackets were the standard errors. The region dummies were included in the analysis but not presented by Kramer et al. [1].

**Table 3:** Multivariate regression coefficients of independent variables predicting log taxable income, log non-work income, log total income, and poverty status for the full sample.

Variables	Log Taxable Income	Log Non-Work Income	Log Total Income	Poverty Status
	Coef.(robust S.E.)	Coef.(robust S.E.)	Coef.(robust S.E.)	Coef.(robust S.E.)
(Male)				
Female	−0.47 ***	1.01 ***	−0.33 ***	−101.15 **
	(0.09)	(0.28)	(0.08)	(37.09)
(White)				
Black	−0.11	−0.04	−0.11	−37.99
	(0.08)	(0.20)	(0.07)	(20.71)
Hispanic	0.01	−0.21	−0.04	−55.52
	(0.13)	(0.28)	(0.11)	(30.77)
Other race	−0.28	−0.86	−0.42	−89.10
	(0.39)	(0.46)	(0.38)	(44.51)
Log number of children	−0.09	0.57 *	−0.01	−44.14
	(0.13)	(0.27)	(0.11)	(43.33)
Age	0.13 **	0.11	0.12 ***	12.29
	(0.04)	(0.08)	(0.03)	(6.57)
Age squared	−0.00 **	−0.00	−0.00 ***	−0.08
	(0.00)	(0.00)	(0.00)	(0.09)
(Divorced)				
Widowed	−0.31	0.15	−0.18	−79.90
	(0.25)	(0.66)	(0.22)	(51.48)
Separated	−0.27 *	−0.03	−0.25 *	−65.11 *
	(0.10)	(0.28)	(0.10)	(29.20)
Never married	−0.04	−0.19	−0.10	5.39
	(0.14)	(0.19)	(0.12)	(43.34)
Years of education	0.01	−0.23	0.02	−43.09
	(0.11)	(0.16)	(0.09)	(30.44)
Years of education squared	0.01	0.01	0.01	3.03 *
	(0.00)	(0.01)	(0.00)	(1.20)
Years of experience	0.09 ***	−0.02	0.06 ***	5.11
	(0.01)	(0.06)	(0.01)	(4.47)
Years of experience squared	−0.00 ***	−0.00	−0.00 **	−0.05
	(0.00)	(0.00)	(0.00)	(0.22)
(Northeast)				
North central	−0.33 *	−0.48	−0.28 **	−68.70 *
	(0.13)	(0.35)	(0.10)	(31.20)
South	−0.30 *	−0.52	−0.31 **	−64.50 *
	(0.12)	(0.30)	(0.11)	(26.46)
West	−0.11	0.01	−0.06	11.26
	(0.12)	(0.37)	(0.11)	(33.16)
(2011)				
2013	−0.04	0.05	−0.08	−24.02 *
	(0.05)	(0.13)	(0.04)	(10.32)
Constant	6.78 ***	5.39 *	7.04 ***	111.04
	(1.16)	(2.15)	(1.04)	(294.53)
F statistic	25.39 ***	4.14 ***	29.29 ***	22.01 ***
*N*	1135	645	1135	1135
Adjusted R^2^	0.37	0.18	0.42	0.37

* *p* < 0.05; ** *p* < 0.01; *** *p* < 0.001.

## Data Availability

The public use dataset analyzed during the current study is available online: Panel Study of Income Dynamics, public use dataset. Produced and distributed by the Survey Research Center, Institute for Social Research, University of Michigan, Ann Arbor, MI.

## Abbreviations

M/P: Means/proportions; NR: Not reported; PSID: Panel Study of Income Dynamics; Coef.: Coefficient; S.E.: Standard error.

## References

[1] Kramer K.Z., Myhra L.L., Zuiker V.S., Bauer J.W. (2016). Comparison of Poverty and Income Disparity of Single Mothers and Fathers Across Three Decades: 1990–2010. Gend. Issues.

[2] Vespa J., Lewis J.M., Kreider R.M. (2013). America’s Families and Living Arrangements: 2012 Population Characteristics. Curr. Popul. Rep..

[3] U.S. Census Bureau America’s Families and Living Arrangements: 2009. https://www.census.gov/population/www/socdemo/hh-fam/cps2009.html.

[4] U.S. Census Bureau Survey of Income and Program Participation Datasets, 2008. https://www.census.gov/programs-surveys/sipp/data/datasets.2008.html.

[5] Corcoran M. (1995). Rags to Rags: Poverty and Mobility in the United States. Annu. Rev. Sociol..

[6] Duncan G.J., Brooks-Gunn J. (1999). Consequences of Growing Up Poor.

[7] McLoyd V.C. (1998). Socioeconomic Disadvantage and Child Development. Am. Psychol..

[8] Rendall M.S. (1999). Entry or Exit? A Transition-Probability Approach to Explaining the High Prevalence of Single Motherhood among Black Women. Demography.

[9] Vartanian T.P., McNamara J.M. (2004). The Welfare Myth: Disentangling the Long-Term Effects of Poverty and Welfare Receipt for Young Single Mothers. J. Sociol. Soc. Welf..

[10] Neblett N.G. (2007). Patterns of Single Mothers’ Work and Welfare Use: What Matters for Children’s Well-Being?. J. Fam. Issues.

[11] Lee S., Oh G.-T., Hartmann H., Gault B. (2004). The Impact of Disabilities on Mothers’ Work Participation: Examining Differences between Single and Married Mothers.

[12] Richard P., Gaskin D.J., Alexandre P.K., Burke L.S., Younis M. (2014). Children’s Emotional and Behavioral Problems and Their Mothers’ Labor Supply. Inq. A J. Med Care Organ. Provis. Financ..

[13] (2016). Richard, P The Burden of Medical Debt Faced by Households with Dependent Children in the United States: Implications for the Affordable Care Act of 2010. J. Fam. Econ. Issues.

[14] Richard P. (2016). Children’s Mental Disorders and Their Mothers’ Earnings: Implications for the Affordable Care Act of 2010. J. Fam. Econ. Issues.

[15] Ziol-Guest K.M. (2009). A Single Father’s Shopping Bag: Purchasing Decisions in Single-Father Families. J. Fam. Issues.

[16] Lin M., Lucas H.C., Shmueli G. (2013). Research Commentary—Too Big to Fail: Large Samples and the p-Value Problem. Inf. Syst. Res..

[17] Lantz B. (2013). The Large Sample Size Fallacy. Scand. J. Caring Sci..

[18] Veldhuizen I., Pasker-De Jong P., Atsma F. (2012). Significance or Relevance: What Do You Use in Large Samples? About p Values, Confidence Intervals, and Effect Sizes. Transfusion.

[19] Panel Study of Income Dynamics (2013). PSID Main Interview User Manual: Release 2013.

[20] Panel Study of Income Dynamics (2011). PSID Main Interview User Manual: Release 2011.

[21] Einolf C.J. (2017). Parents’ Charitable Giving and Volunteering: Are They Influenced by Their Children’s Ages and Life Transitions? Evidence From a Longitudinal Study in the United States. Nonprofit Volunt. Sect. Q..

[22] Marcoulides K.M., Grimm K.J. (2016). Data Integration Approaches to Longitudinal Growth Modeling. Educ. Psychol. Meas..

[23] Stokes J.E. (2017). Do ‘His’ and ‘Her’ Marriages Influence One Another? Contagion in Personal Assessments of Marital Quality among Older Spouses over a Four-Year Span. Soc. Psychol. Q..

[24] Gonalons-Pons P., Schwartz C.R. (2017). Trends in Economic Homogamy: Changes in Assortative Mating or the Division of Labor in Marriage?. Demography.

[25] Eng J. (2003). Sample Size Estimation: How Many Individuals Should Be Studied?. Radiology.

[26] Bhave D.P., Amit K., Glomb T.M. (2013). Pay Satisfaction and Work–Family Conflict across Time. J. Organ. Behav..

[27] Lievens F., Sanchez J.I., Bartram D., Brown A. (2010). Lack of Consensus among Competency Ratings of the Same Occupation: Noise or Substance?. J. Appl. Psychol..

[28] Meyer B.D., Sullivan J.X. (2004). The Effects of Welfare and Tax Reform: The Material Well-Being of Single Mothers in the 1980s and 1990s. J. Public Econ..

[29] U.S. Census Bureau Poverty Thresholds by Size of Family and Number of Children, 2019. https://www.census.gov/data/tables/time-series/demo/income-poverty/historical-poverty-thresholds.html.

[30] Bauer J.W., Braun B., Olson P.D. (2000). Welfare to Well-Being Framework for Research, Education, and Outreach. J. Consum. Aff..

[31] U.S. Bureau of Labor Statistics Consumer Price Index, 2019. https://www.bls.gov/cpi/.

[32] Zhan M., Pandey S. (2004). Postsecondary Education and Economic Well-Being of Single Mothers and Single Fathers. J. Marriage Fam..

[33] U.S. Census Bureau How the Census Bureau Measures Poverty, 2019. https://www.census.gov/topics/income-poverty/poverty/guidance/poverty-measures.html.

[34] Wolfe R., Carlin J.B. (1999). Sample-Size Calculation for a Log-Transformed Outcome Measure. Control. Clin. Trials.

[35] Mincer J. (1974). Schooling and Earnings. Schooling, Experience, and Earnings.

[36] StataCorp (2015). Stata Statistical Software: Release 14.

[37] King M., Steven R., Alexander J.T., Flood S., Genadek K., Schroeder M.B., Trampe B., Vick R. (2010). Integrated Public Use Microdata Series, Current Population Survey: Version 3.0.

[38] Douthitt R.A. (2000). ‘Time to Do Chores?’ Factoring Home-Production Needs into Measures of Poverty. J. Fam. Econ. Issues.

[39] Livingston G. (2013). The Rise of Single Fathers A Ninefold Increase Since 1960. https://www.pewsocialtrends.org/2013/07/02/the-rise-of-single-fathers/.

[40] Grall T. (2013). Custodial Mothers and Fathers and Their Child Support: 2011. https://www.census.gov/library/publications/2013/demo/p60-246.html.

